# The Impact of Technology Adaptation on Academic Engagement: A Moderating Role of Perceived Argumentation Strength and School Support

**DOI:** 10.3389/fpsyg.2022.962081

**Published:** 2022-07-07

**Authors:** Jing Zhao, Muhammad Awais-E-Yazdan, Iqra Mushtaque, Limei Deng

**Affiliations:** ^1^College of Foreign Studies, Hubei Normal University, Huangshi, China; ^2^College of Business, Universiti Utara Malaysia, Sintok, Malaysia; ^3^Bahadur Sub Campus Layyah, Bahauddin Zakariya University, Layyah, Pakistan; ^4^College of Education Science, Hubei Normal University, Huangshi, China

**Keywords:** academic engagement, school support, argumentative strength, technology adaptation, China

## Abstract

The COVID-19 pandemic has impacted routine activities such as attending to school and transferring education online. This study explores students’ perceptions of technology adoption and academic engagement using data from a survey (*N* = 465), with perceived argumentation and school support serving as moderators. The data were collected using a convenience sampling technique. The authors examined the association between perceived utility, perceived digital competitiveness, and perceived ease of use and academic engagement. While perceived utility and ease of use of online learning technologies do not appear to be connected with academic engagement, digital competence is. It is argued that there is a need to introduce an improvised mechanism for technology in schools. Academic involvement has no effect on perceived reasoning power, but social support has a considerable effect on academic engagement.

## Introduction

The COVID-19 pandemic has compelled the education sector to seek alternatives to traditional classroom instruction. As a result, teachers and students have used online learning in novel ways, and most universities in China have turned to online learning as a method for the first time (Mushtaque et al., 2021). Regardless of how well-prepared educators in elementary and secondary schools are, the quick shift to online learning is a reality (El-Sayad et al., 2021). Online classrooms were launched after the Chinese government declared a state of emergency. Every educational institution has made an effort to help students study more effectively. Their main goal was to organize and execute relevant content in a virtual setting, as well as to address student issues. The report is timely given the current state of affairs in China, where both online education and offline education are harming pupils. In many cases, academic engagement of students with online learning is the major goal. Mandatory online instruction is not the same as an unprompted act during a pandemic, and this has increased educators’ technological-related stress (Mushtaque et al., 2021).

Perception and belief can be used to forecast how people will interact with technology in the future. The Technology Acceptance Model (Fishbein and Ajzen, 1975) demonstrates how individuals think about technology acceptance. TAM can assist in elucidating why individuals believe technology is beneficial and how simple it is to utilize in online education (Liu and Peng, 2005). TAM is better suited for use in online environments because it focuses on how information systems can be used to apply the concepts of ease of use and utility to how people use those (Rini and Khasanah, 2021). In all the mediums of instruction, student engagement is very important in online courses for students to learn and have learning satisfaction. Student engagement refers to “the student’s psychological commitment in academic work as well as effort given to mastering the knowledge and talents that academic work is designed to nurture” (Lane and Perozzi, 2014). Students are more likely to use an e-learning system if they believe it is beneficial to their studies. There are still some students who have reservations about using online learning (Yeou, 2016).

We are particularly interested in students’ perceptions of an online course’s e-usability, content, and how well it prepares them for the final exam. So, perceived usefulness is “the degree to which a person believes that utilizing a given technology will increase their academic performance” (Davis, 1989). A study’s findings show that e-learning is beneficial when: (1) the teacher is engaged and their activities in an e-course directly affect the student’s attitude toward the subject and the lecturer’s classroom performance (Granić and Marangunić, 2019); (2) technology acceptance indirectly affects the student’s attitude toward the subject and the lecturer’s classroom performance (Hanham et al., 2021); and (3) technology acceptance directly affects the student’s attitude toward the subject and lecturer’s classroom performance (Keržič et al., 2019). Students must have a learning aptitude toward digital learning that would prompt them to engage in digital-based learning activities. Within to put it another way, digital competence refers to the ability and willingness of students in higher education to use digital technology to achieve their educational goals and objectives (Hong and Kim, 2018). Some academicians believe that digital competence can increase students’ academic engagement in digital learning environments (Bergdahl et al., 2019; Basantes-Andrade et al., 2020). So, it is important to have the right technology, skills, and the ability to deal with unexpected problems when using technology in education. Mandated online education was put in place because schools were going to close, it clearly needs help from the school (Bao, 2020), and teachers play an important role in helping students make digital learning more meaningful. In any classroom, getting students to participate actively can be a challenge. Teachers face significant challenges (and opportunities) when it comes to encouraging and evaluating student engagement in online courses this year because of the shift to online teaching (Majidi et al., 2021). The use of argumentation has been found to have a significant impact on student outcomes. Students who would not ordinarily have the opportunity to speak up in class were found to become more engaged as a result of the argumentation method being used in those classrooms (Polat et al., 2016).

There has been a lot of research on COVID-19’s effect on higher education in countries like the United States and Europe (Johnson et al., 2020). However, because COVID-19 came so quickly, many higher education institutions in less-developed countries were unprepared and did not know much about the benefits of online learning. Countries that are still developing have a wide range of economic, technological, social, cultural, and educational settings (Awais-e-Yazdan and Hassan, 2020). There is a lot of interest in online learning in Arab countries like Egypt and Saudi Arabia, but it is still in its early stages there (Adel, 2015). Educational capabilities improve as technology progresses. Online education can be used for both differentiated/remedial training and educational discovery. The present study is critical to analyzing students’ academic involvement and the elements that contribute to their perceived usefulness toward online learning, digital competency, perceived argumentation strength, and school support beyond the COVID-19 epidemic in developed countries like China. It enables legislators to comprehend students’ perceptions when online teaching techniques are imposed and to make appropriate adjustments to students’ education.

## Literature Review and Hypothesis Development

### Perceived Usefulness and Academic Engagement

Using technology helps students be more involved, and it has been shown to have a positive effect on student behavior like online academic engagement, which is why it is important for teachers to use it (Rashid and Asghar, 2016). Another study revealed that students’ perceptions of how much time they spend on online assignments and activities may be affected by how useful the online learning system is, and that this relationship should be looked into more (Burch et al., 2017). Thus, in online learning contexts, perceived usefulness might show how students think online learning is good for them. Previous research has shown that online students who think a course is useful are more likely to participate in MOOCs, game-based learning, and blended learning (Jung and Lee, 2018; Ab Rahman et al., 2019). When Fisher and other people did this, they said that (2018). Perceived value also plays a vital role in how satisfied students are with online learning mode (Fisher et al., 2018; Hui et al., 2019). Furthermore, there is a strong correlation between student engagement and outcomes in online programs, especially when it comes to student perception about usefulness and satisfaction (Bolliger and Halupa, 2018). Similarly, another study revealed that student participation has a substantial impact on student learning and satisfaction in online learning environments (Wells and Council, 2010).

### Digital Competence and Academic Engagement

There are a lot of different ways to study digital competency (van Laar et al., 2017). As Calvani et al. (2012) say, the three parts of digital competence are technical skills, cognitive skills, and ethical awareness. They say that technical literacy is a mix of visual literacy, technical idea understanding, and the ability to apply that knowledge in real-world situations, which they call technical literacy. Academic engagement is closely linked to digital competency, but there is not a lot of empirical evidence to back it up. It has been studied in the past, but only indirectly. For example, (Kim et al., 2018) found that students did not change their participation based on how well they thought they were at using technology and how positive they thought they were. When learning agility was used as a mediator, students were more likely to be engaged and more likely to be able to use digital tools. On the other hand, researcher found that students with a higher level of digital competence were more interested in using digital technology. Practicing professional engagement can help teachers improve their digital skills (Bergdahl et al., 2019; Reisoğlu and Çebi, 2020).

### Perceived Ease of Use and Academic Engagement

Students’ acceptance of online learning as a new study platform might be influenced by perceived ease of use. The term “perceived ease of use” refers to whether or not students believe the platform is simple to use. The system’s perceived ease of use is consistent with (Davis, 1989) concept of perceived ease of use. Another study investigated the student acceptability of e-learning and identified factors that influenced it at a Malaysian teacher education institute. This implies that perceived ease of use influences students’ acceptance of e-learning as a convenience factor (Taat and Francis, 2019). Furthermore, it was predicted that after online face-to-face learning, students would use e-learning to self-learn. This enables students to easily access shared course materials and participate in online learning. Perceived ease of use influences students’ attitudes about online learning (Johari et al., 2015). A researcher studied the variables of student acceptability of online learning and investigated how they can lead to student intention to use it. As a result, pupils did not consider online learning to be simple. Students say that online education is difficult to use (Farahat, 2012). According to research findings, students’ opinions of online learning and the social influence of their referent groups have a significant impact on their desire to engage in it. Additionally, students’ social influence, perceived ease of use, perceived utility, and attitudes toward online learning can be utilized to predict their proclivity for online learning (Utami, 2021).

### Perceived Argumentation Strength and Academic Engagement

Academic engagement has a significant effect on both students’ short-term success and the long-term quality of the learning they accomplish. Teachers can use cumulative arguments to pique students’ interest in academics by having students explain and justify their ideas and then present them to the class as a whole (Brown, 2017). Learning to debate effectively can help you think more clearly, solve problems more effectively, make more informed decisions, and broaden your knowledge base (Osborne et al., 2004). Educational academics have traditionally focused on math and science arguments, but they have increasingly begun to focus on social science and humanities arguments as well. Despite evidence that this deepens student involvement in the learning process, few studies have looked at how students assess arguments (Lu and Zhang, 2013). Students can now rate their peers’ work online, allowing them to compare their own feedback to that of the teacher. Additionally, online platforms enable teachers to develop argument evaluation rubrics for their students.

### School Support and Academic Engagement

In the past, it has been demonstrated that supportive relationships, classmates, teachers, and parents boost students’ educational experiences and involvement (Xerri et al., 2017). It is not surprising, given that the level of support obtained from prominent individuals in these many social circumstances has a real impact on someone’s advancement. This is particularly true throughout adolescence, when social ties play a critical role in molding youth experiences (Ferrer-Wreder et al., 2008; Vargas-Madriz and Konishi, 2021). The term “school belonging” refers to the degree to which children feel accepted, respected, and supported in school by their teachers and peers. As a result, this concept conveys feelings of inclusion in the life and activities of the school. According to previous research, school belonging may have a relationship between social support and academic involvement (Zumbrunn et al., 2014). While online learning students faced minimal connection with teachers and classmates throughout the COVID-19 period, the current study will investigate the quality of school support obtained by students while running online learning modules.

### Conceptual Framework of the Study

**Figure 1:**
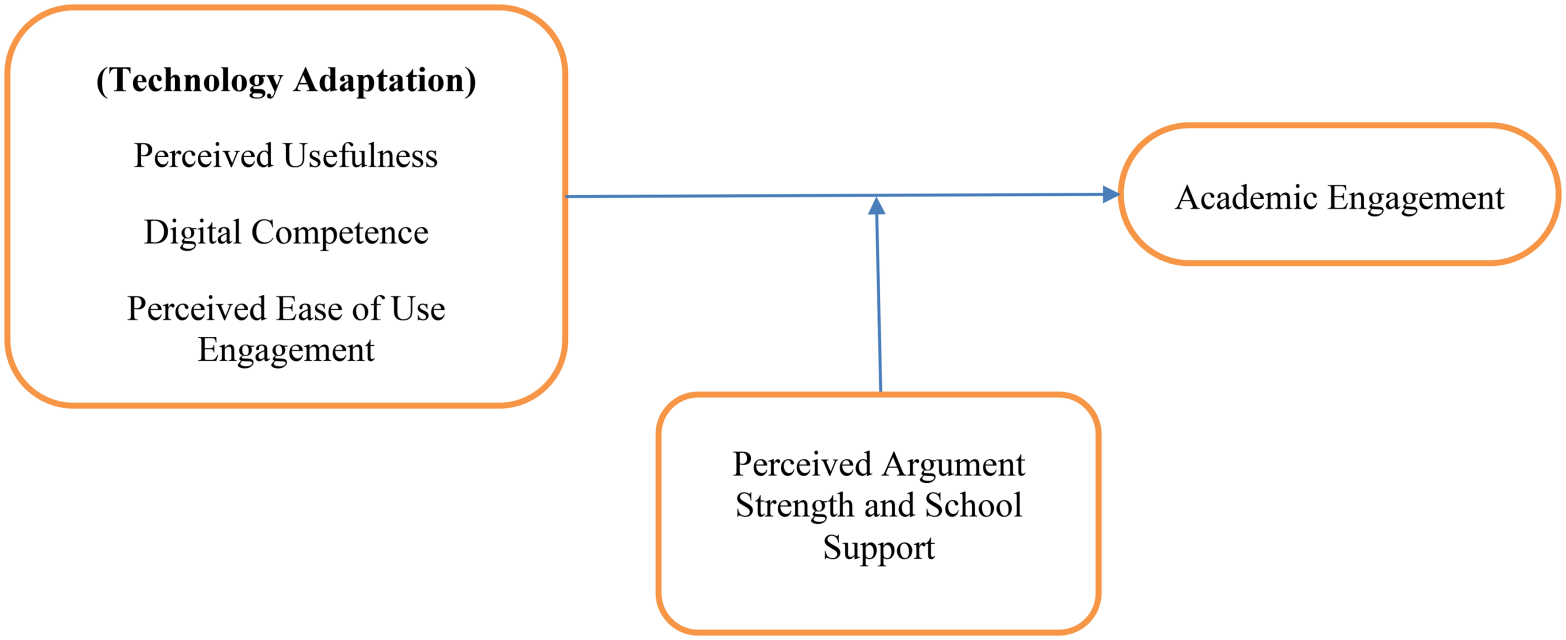
Conceptual model of the research.

Figure 1 shows the conceptual model regarding the predictors of students’ academic engagement in online learning during the COVID-19 period is linked. Determinants are perceived usefulness and digital competence, perceived easy to use, perceived argument strength, and school support.

### Study Hypothesis

*H1*: Perceived usefulness has a significant relationship with academic engagement among secondary school students.

*H2*: Digital Competence has a significant relationship with academic engagement among secondary school students.

*H3*: Perceived ease of use has a significant relationship with academic engagement among secondary school students.

*H4*: Perceived argumentation strength moderates the relationship between perceived usefulness and academic engagement among secondary school students.

*H5*: School support moderates the relationship between perceived usefulness and academic engagement among secondary school students.

## Materials and Methods

### Research Questionnaire and Methodology

The current research tools were the self-reported questionnaire. We adapted Lee et al. (2005)‘s technology acceptance model and assessment scales for perceived usefulness (3 items) and perceived ease of use for engagement (3 items) purposes because the authors had already pre-tested and verified their application to e-learning activities. Similar to the original design, the items were rated on a 7-point Likert scale (Lee et al., 2005). Researchers used the digital competency framework to construct a digital competency questionnaire that was then used to gather data. From 1 (“strongly disagree”) to 5 (“strongly agree”), respondents could answer on a 5-point Likert scale. There are a total of five items included in this study (Casillas Martín et al., 2019). To assess the level of school support, the students’ responses were measured using the Lam et al. (2010) scale. It has four items. Students’ argument strength of individuals’ perceptions of the message’s content was determined. Zhao et al. developed a six-item measure in which participants could respond 1 to 7 (strongly disagree to strongly agree; Zhao et al., 2011). We measured academic engagement by using Fredricks et al. (2004) scale. It has 12 items, but in the current study, we used only eight items because online academic engagement was the main purpose of the study. A study has shown scales to be valid and reliable (Martin, 2009).

### Participants of the Research

The target population of this study includes school students in Hubei, China. The students studying in schools located in Hubei, China, were targeted by incorporating convenience sampling technique. Convenience sampling technique is a particular type of non-probability technique that depends on the participants which are easily accessible to take part in the research from the population. With the help of convenience sampling, the researchers can easily obtain data from the targeted population. The Chinese Ministry of Education implemented a number of safety measures to minimize student clustering during the COVID-19 outbreak. For example, the spring 2020 school session has been delayed by 2 weeks. When the semester began, teachers educated their students online about the possibility of a school closure. Criteria for instruction varied according to school and classroom. Others made video tools available to students at a later date. Online education methods such as asynchronous, synchronous, and hybrid were used. After receiving approval from our study institute’s institutional review board, we shared the surveys on numerous educational Facebook and WhatsApp groups. According to the guidelines, the target market was secondary students who used online education as a result of the COVID-19 pandemic. We did not award completion points to deter repeated submissions. One questionnaire took approximately 10 to 15 min to complete.

## Results

### Assessment of Measurement Model

**Figure 2:**
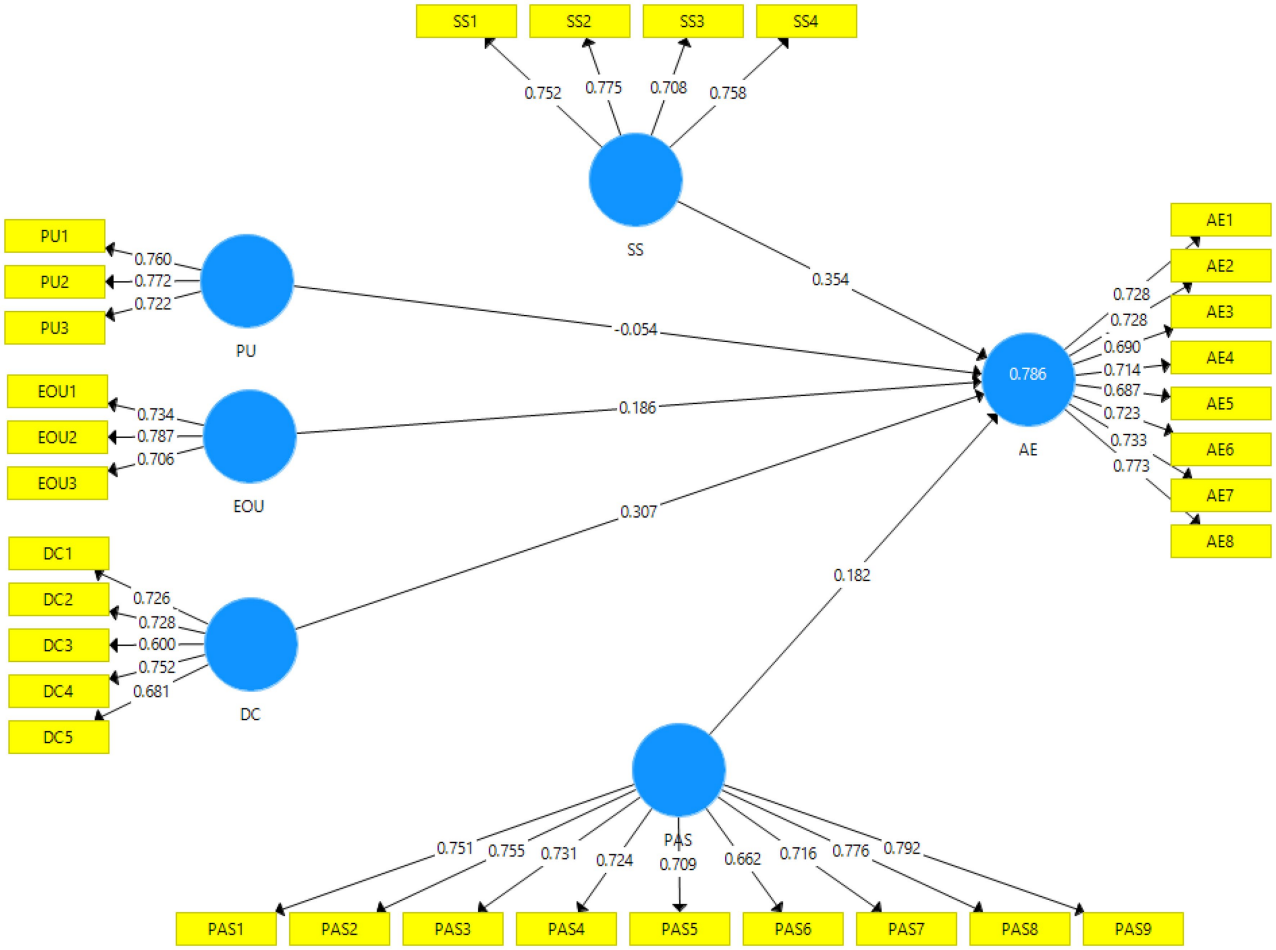
Measurement model.

The measurement model which is also known as an outer model is followed to evaluate individual item reliability, convergent validity, internal consistency reliability, and discriminant validity. The elements of the measurement model are listed in Figure 2.

### Reliability Analysis

The present study determined individual item reliability by calculating the factor loading of every construct (Duarte and Raposo, 2010; Hair et al., 2016). As recommended by Hair et al. (2016), items along with factor loadings above 0.40 can be retained. It is further recommended that items should be removed if its removal can increase the values of composite reliability (CR) and average variance extracted (AVE; Hair et al., 2016; Awais-E-Yazdan et al., 2022). The study retained each item of constructs as the removal did not increase factor loadings.

### Internal Consistency Reliability

Internal consistency is the correlation among the different items which are intended to determine a similar construct. Internal consistency of constructs can be evaluated by composite reliability (CR) and Cronbach’s alpha (Hair et al., 2017). In the latest studies, internal consistency reliability is measured by CR as compared to Cronbach’s alpha. Hence, the current study adopted CR to measure internal consistency reliability (Barroso et al., 2010). Values below 0.60 are not acceptable; values between 0.60 and 0.70 show average internal consistency reliability, and values between 0.70 and 0.90 signify adequate internal consistency reliability (Hair et al., 2011). The adequate internal consistency reliabilities of construct are listed in Table 1.

**Table 1:** Factor loadings, composite reliability, and average variance extracted.

Constructs	Items	Loadings	CR	AVE
Perceived usefulness	PU1	0.760	0.796	0.565
	PU2	0.772		
	PU3	0.722		
Digital Competence	DC1	0.726	0.826	0.511
	DC2	0.728		
	DC3	0.600		
	DC4	0.752		
	DC5	0.681		
Perceived ease of use	EOU1	0.734	0.787	0.552
	EOU2	0.787		
	EOU3	0.706		
Perceived argumentation strength	PAS1	0.751	0.914	0.542
	PAS2	0.755		
	PAS3	0.731		
	PAS4	0.724		
	PAS5	0.709		
	PAS6	0.662		
	PAS7	0.716		
	PAS8	0.776		
	PAS9	0.792		
School support	SS1	0.752	0.836	0.560
	SS2	0.775		
	SS3	0.708		
	SS4	0.758		
Academic engagement	AE1	0.728	0.897	0.522
	AE2	0.128		
	AE3	0.690		
	AE4	0.714		
	AE5	0.687		
	AE6	0.723		
	AE7	0.733		
	AE8	0.773		

### Convergent Validity

Average variance extracted (AVE) was used to determine convergent validity (Hair et al., 2009). By measuring a proper AVE the value of each construct must be greater than 0.50 (Fernandes, 2012; Hair et al., 2014). Table 1 shows the appropriate values of AVE.

### Discriminant Validity

The degree to which a construct is distinguished from other constructs is known as discriminant validity (Hair et al., 2017). On the basis of AVE values, Fornell and Larcker’s (1981) criterion is incorporated to evaluate discriminant validity. In addition, discriminant validity is also measured by cross-loadings (Grégoire and Fisher, 2006). Accordingly, the loading of every indicator must be greater than its cross-loadings with other indicators. Moreover, the heterotrait-monotrait ratio of correlations (HTMT) is another method used to establish proper discriminant validity (Henseler et al., 2015). It is a factor correlation that differentiates between two factors (Henseler et al., 2016). Table 2 shows the Fornell-Larcker criterion, Table 3 displays cross-loadings, and Table 4 shows the HTMT results of this study.

**Table 2:** Latent variable correlations and square roots of average variance extracted (AVE).

	AE	DC	EOU	PAS	PU	SS
AE	**0.723**					
DC	0.640	**0.714**				
EOU	0.713	0.657	**0.743**			
PAS	0.719	0.693	0.649	**0.736**		
PU	0.683	0.685	0.741	0.654	**0.752**	
SS	0.626	0.633	0.721	0.673	0.666	**0.749**

*Entries in the boldface represent the square root of average variance extracted (AVE), AE*, *Academic engagement; DC, Digital competence; EOU, Ease of Use; PAS, Perceived argumentative strength; PU, Perceived usefulness; and SS, Social support*.

**Table 3:** Cross loadings.

	AE	DC	EOU	PAS	PU	SS
AE1	**0.728**	0.610	0.562	0.525	0.536	0.596
AE2	**0.728**	0.627	0.565	0.471	0.605	0.618
AE3	**0.692**	0.550	0.585	0.529	0.494	0.561
AE4	**0.714**	0.629	0.580	0.445	0.577	0.572
AE5	**0.687**	0.594	0.587	0.519	0.554	0.584
AE6	**0.723**	0.608	0.569	0.571	0.554	0.581
AE7	**0.733**	0.595	0.557	0.486	0.604	0.643
AE8	**0.773**	0.639	0.581	0.603	0.596	0.616
DC1	0.571	**0.726**	0.535	0.458	0.697	0.630
DC2	0.590	**0.728**	0.542	0.476	0.696	0.605
DC3	0.519	**0.600**	0.584	0.467	0.517	0.551
DC4	0.650	**0.752**	0.689	0.576	0.593	0.525
DC5	0.595	**0.681**	0.638	0.438	0.589	0.611
EOU1	0.565	0.595	**0.734**	0.470	0.533	0.572
EOU2	0.633	0.701	**0.787**	0.561	0.578	0.528
EOU3	0.568	0.609	**0.706**	0.410	0.558	0.582
PAS1	0.480	0.439	0.407	**0.751**	0.416	0.461
PAS2	0.527	0.512	0.486	**0.755**	0.477	0.482
PAS3	0.468	0.500	0.436	**0.731**	0.483	0.468
PAS4	0.460	0.430	0.380	**0.724**	0.433	0.449
PAS5	0.547	0.570	0.533	**0.709**	0.530	0.537
PAS6	0.613	0.560	0.546	**0.662**	0.495	0.522
PAS7	0.616	0.585	0.610	**0.716**	0.585	0.615
PAS8	0.485	0.480	0.396	**0.776**	0.458	0.446
PAS9	0.487	0.444	0.418	**0.792**	0.391	0.410
PU1	0.557	0.608	0.595	0.494	**0.760**	0.675
PU2	0.645	0.705	0.540	0.509	**0.772**	0.597
PU3	0.556	0.678	0.559	0.470	**0.722**	0.597
SS1	0.596	0.622	0.617	0.494	0.661	**0.752**
SS2	0.617	0.677	0.548	0.512	0.735	**0.775**
SS3	0.633	0.644	0.529	0.497	0.653	**0.708**
SS4	0.623	0.548	0.559	0.511	0.542	**0.758**

*DC, Digital competence; EOU, Ease of Use; PAS, Perceived argumentative strength; PU, Perceived usefulness; and SS, Social support.*

**Table 4:** HTMT correlation matrix for discriminant validity.

	AE	DC	EOU	PAS	PU	SS
AE	–					
DC	0.750	–				
EOU	0.352	0.735	–			
PAS	0.489	0.838	0.869	–		
PU	0.850	0.658	0.818	0.867	–	
SS	0.651	0.722	0.619	0.583	0.816	–

### Assessment of Measurement Model

**Figure 3:**
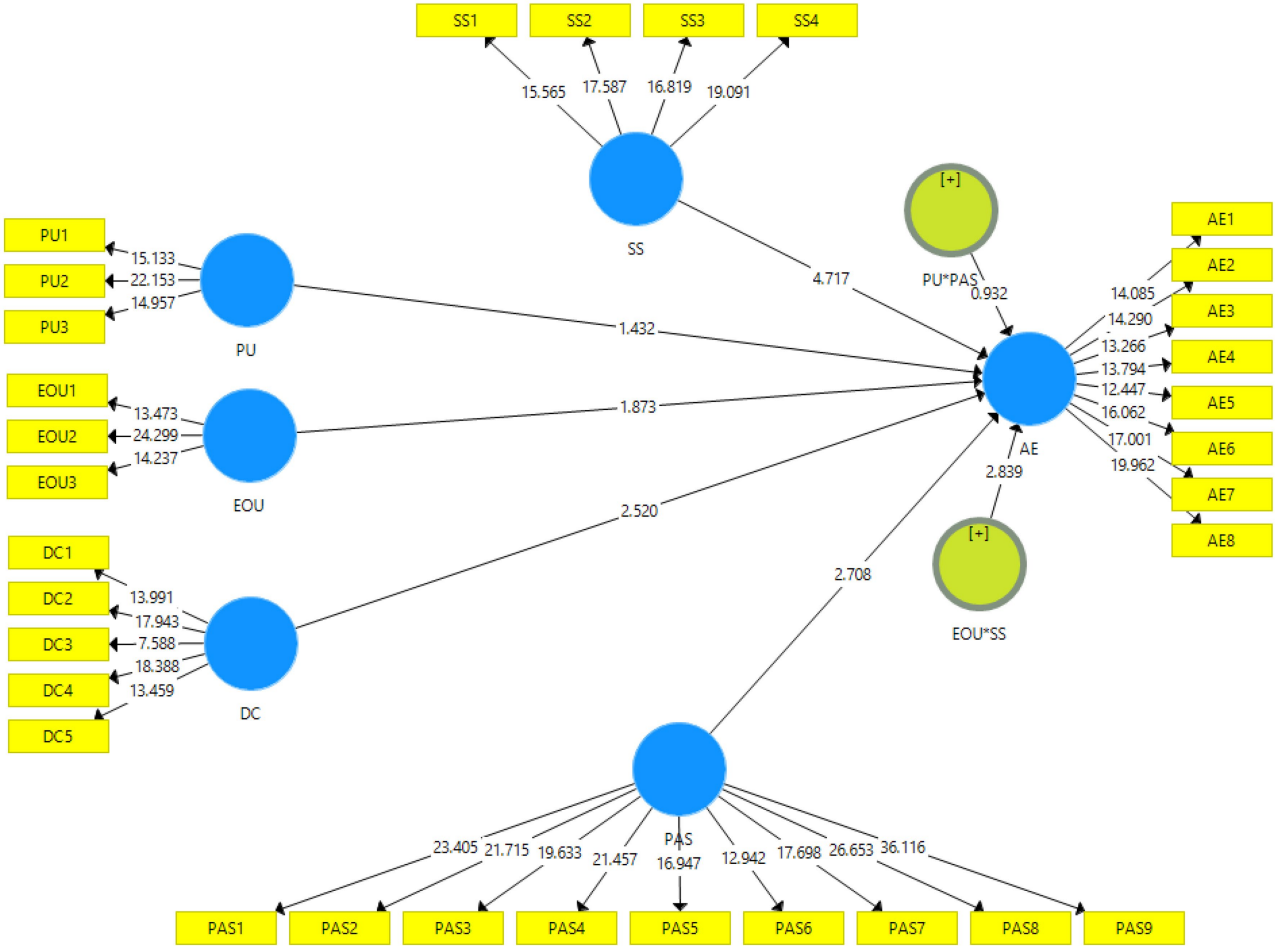
Bootstrapping.

This study used the standardized bootstrapping process with 5,000 bootstrap samples and 465 samples to measure the path coefficients’ significance. Figure 3 shows the measurements of the structural model (direct relationship and moderating effect).

#### Structural Model (Direct Relationship and Moderating Effect)

The structural model depicts the path coefficients of the hypothesized relationships. Hypothesis H1 states that “Perceived usefulness has significant relationship with academic engagement among secondary school students.” The results in Figure 3 and Table 5 showed a non-significant relationship between perceived usefulness and academic engagement (*β* = 0.097; *t* = 1.432; *p* > 0.153). Hypothesis H2 states that “Digital competence has significant relationship with academic engagement among secondary school students” The results indicate that Digital competence has a significant and positive relationship with academic engagement (*β* = 0.194; *t* = 2.520; *p* < 0.012). Similarly, hypothesis H3 states that “Perceived ease of use has significant relationship with academic engagement among secondary school students.” The results in Figure 3 and Table 5 showed that there is non-significant relationship between perceived ease of use and academic engagement (*β* = 0.112; *t* = 1.873; *p* > 0.062). Moreover, hypothesis H4 states that “Perceived argumentation strength moderates the relationship between perceived usefulness and academic engagement among secondary school students.” The result indicated that there is no moderation between perceived argumentation strength and academic engagement (*β* = 0.095; *t* = 0.932; *p* > 0.352). Lastly, hypothesis H5 states that “School support moderates the relationship between perceived ease of use and academic engagement among secondary school students. The result indicated that school support positively moderates the relationship between perceived ease of use and academic engagement (*β* = 0.256; *t* = 2.839; *p* < 0.005).

**Table 5:** Structural model assessment with interactions.

Hypothesis	Relationships	Beta	SE	T-Value	value of *p*	Decision
H1	PU→AE	0.097	0.068	1.432	0.153	Not supported
H2	DC→AE	0.194	0.077	2.520	0.012	Supported
H3	PEU→AE	0.112	0.060	1.873	0.062	Not supported
H4	PU*PAS→AE	0.095	0.102	0.932	0.352	Not supported
H5	EOU*SS→AE	0.256	0.090	2.839	0.005	Supported

*DC*, *Digital competence; EOU, Ease of Use; PAS, Perceived argumentative strength; PU, Perceived usefulness; and SS, Social support*.

## Discussion

Technology has evolved into a major source of innovation and efficiency improvement on a global scale. For both inside and outside the classroom, technology has become an important part of the learning process for children. Students and teachers in secondary schools place a high priority on technology. In the current study, the researchers wanted to know how effective e-learning is to students and how engaged they are in online classes during the COVID-19. The result revealed that perceived usefulness and perceived ease of use toward academic engagement has a non-significant association. The results are aligned with the finding of an ingenious study, in which students revealed that there is a scarcity of online study materials, as well as no or slow internet access and electrical load shedding. As a result of these challenges, student participation and proxy attendance in the online class is poor or non-existent. Students’ health suffers as a result of taking online classes. Poor visions, obesity, sleep deprivation, and behavioral concerns plagued the students (Noor et al., 2020; Mushtaque et al., 2021). China is a developed country in which for the first time, an online learning medium was utilized during the period of COVID-19 to resume educational activities. Even teachers were reluctant to use online modalities (Mushtaque et al., 2021). In terms of “Perceived Ease of Use of E-Learning,” the study discovers that students believe that e-learning platforms are difficult to use and that they are unable of engaging in academic activities. Rural students in underdeveloped nations continue to have a need for inexpensive, durable equipment. In China, due to economic difficulty, the government has ordered a 20% cut in school fees. Regrettably, the government continues to overlook education. Shortsighted lobbyists appear to be grabbing sensitive regions of human progress rather than fostering non-discriminatory inquiry in the virtual environment (Ramani, 2015; Basheer, 2020).

Due to the convenience of smartphones in China, teachers and students choose WhatsApp and Zoom sessions over the other available online learning options. Almost half (40%) of participants used WhatsApp to learn about the COVID-19 outbreak. Teachers and parents commonly use WhatsApp, which is immediately available on all devices. Teachers can easily submit homework or tasks on WhstApp. WhatsApp is the most widely used free messaging program. Teachers can receive photographs and videos from their students immediately. In comparison to other apps, WhatsApp is extremely simple to use, and parents can simply monitor their children’s actions. WhatsApp is popular among educators due to its ease of use, accessibility, high level of student-teacher interaction, and ability to learn anywhere and at any time (Rehman et al., 2021).

On the scale of digital competence, students revealed they feel competence to use online modalities. According to the current study, academic involvement was correlated with school assistance in terms of pandemic-related study resources. This finding corroborates prior research that discovered favorable connections in non-pandemic situations (Bakker et al., 2014; Lesener et al., 2020). The discovered positive link, on the other hand, is consistent with recent reports. According to Elmer et al. (2020), a lack of social support or social isolation during the COVID-19 epidemic increased the chance of developing mental health problems, which can have a direct effect on student engagement. Additionally, our research revealed an association between digital competency and academic engagement. Two points stand out in this regard. On the one hand, the effect was associated with the use of digital teaching tools; on the other hand, it was associated with the use of usable forms. With the COVID-19 epidemic imminent, a recent study concludes that while digital teaching technologies are critical, they must be adopted with students’ needs and digital literacy in mind (Hughes et al., 2020). Additionally, it was observed that the variety of effective learning formats is significant, with more formats being associated with academic engagement. Students desire flexibility and alternatives in their educational practices in order to maintain control in an uncertain and frightening pandemic environment (Rippé et al., 2021).

In the current study, perceived argumentative strength was measured as a moderator; according to our best knowledge, it was measured first time with the academic engagement. In our study, perceived argumentative strength has the non-significant association with the academic engagement. It is because, in China, at the secondary level, the WeChat and Zoom media were used to communicate with students. They were not allowed to ask questions or hold open discussions about the course material. In lower income countries, there is still a need to invest in technology setup in educational institutions and also a need to conduct workshops for teachers as well as for students on the utilization of technology.

## Study Implications

The major theoretical gap addressed in the present study is the moderating effect of perceived argumentation strength and school support in describing the relationship between technology adaptation and academic engagement in Chinese schools. Therefore, the research presents a considerable theoretical contribution to the body of knowledge in the area of education. The study empirically justified its importance and describe its significance to the education sector. Likewise, the study extended the body of education literature by examining this extensive and unique model. Moreover, this is the first empirical piece of research which incorporated two moderating variables, namely perceived argumentation strength and school support on the relationship between technology adaptation and academic engagement in the Chinese education sector. Besides this, the empirical findings of the current study show significant practical implications to the Chinese education sector in order to improve online and offline education. The empirical results illustrated that the level of academic engagement among Chinese students is adequate. Thus, the concerned authorities must put efforts toward the education sector of China. Our findings state that digital competence has a significant and positive relationship with academic engagement among secondary school students. It means that digital competence can help to extend academic engagement among school students. Although, managers have a significant role here to replicate those aspects which directly influence the academic engagement. Similarly, the results indicated that school support positively moderates the relationship between perceived ease of use and academic engagement among secondary school students. Here, the individuals should know that how much school support is essential in order to engage more students in academic activities. However, it is exposed from the study findings that how much the support culture enhances the engagement level of the students.

## Limitations and Future Directions

A few limitations of the study are presented for future studies. First, the data were collected with the help of questionnaire using cross-sectional study design due to time constraints. To overcome this issue, in future, longitudinal study design should be followed in order for the further validations. Second, the study is conducted in Hubei, China. Therefore, the findings are limited to this geographical region. Future studies should gather data from other regions or countries in order to increase the generalizability of the results. The third limitation of the study is that we include perceived argumentation strength and school support as moderating variables. Future studies should use other variables such as trust and satisfaction to examine their effects. Likewise, future studies could also use mediator between technology adaptation and academic engagement. Last, this study has targeted the school students. Future studies could examine college and university students also. Additionally, this study is quantitative in nature. Therefore, future studies can follow qualitative or mixed-method approach.

## Conclusion

The study examines secondary school students’ perspectives of e-learning in the context of the continuing COVID-19 outbreak. According to the survey, students prefer e-learning since it enables them to connect with their teachers, friends, and study materials while remaining at home and at their own pace. One of the primary reasons students do not engage in academic engagement is a lack of convenient access to study materials. Because online courses lacked discussion opportunities, it was impossible to argue. School support and flexible class times help students be more involved in their studies. Accordingly, e-learning technologies impede students’ access to information in impoverished nations such as America. This conclusion is supported by e-utility, self-efficacy in learning, convenience of use, and student behavior. Students, according to the poll results, prefer to engage with one another using online learning tools. Additional educators of COVID-19, on the other hand, support online education, and nations such as China are prepared to implement reliable, cost-effective, and secure online education systems. The amount of time, resources, and coordinated efforts expended by stakeholders can determine whether the situation is a blessing or a misfortune.

## Data Availability Statement

The raw data supporting the conclusions of this article will be made available by the authors, without undue reservation.

## Ethics Statement

The studies involving human participants were reviewed and approved by Hubei Normal University, Huangshi, Hubei, China. The patients/participants provided their written informed consent to participate in this study.

## Author Contributions

JZ and MA-E-Y proposed the research idea, analyzed the results, and wrote the manuscript. LD and IM carried out the methodology and extensively edited the manuscript. All authors contributed to the article and approved the submitted version.

## Funding

Teaching Reform on Ideological and Political Education Project of Hubei Normal University: Research on the Design and Practice of Ideological and Political Education in College English Courses Based on ADDIE Model (Project no: KCSZY202149) and Youth Project of Hubei Normal University: Research on the realization path and evaluation of the effect of “two-line” integrated teaching model in primary and secondary schools in the post-epidemic era (Project no: HS2020QN003).

## Conflict of Interest

The authors declare that the research was conducted in the absence of any commercial or financial relationships that could be construed as a potential conflict of interest.

## Publisher’s Note

All claims expressed in this article are solely those of the authors and do not necessarily represent those of their affiliated organizations, or those of the publisher, the editors and the reviewers. Any product that may be evaluated in this article, or claim that may be made by its manufacturer, is not guaranteed or endorsed by the publisher.
